# Institutional Questions

**Published:** 1900-12-29

**Authors:** 


### INSTITUTIONAL QUESTIONS.

### EMERGENCY CARDS.

"Puzzled" asks: "Where can one procure what used to be called 'Emergency Cards'; printed cards to hang up in private houses, schools, &c., giving information as to simple remedies, &c., and advice on 'what to do till the doctor comes'? I have tried in several places lately, and cannot learn where to find them."

Apply to the National Health Society, 53 Berners Street, W. We believe this society issues such cards; but, at any rate from them you would be sure to obtain information as to where they may be procured. The Ladies' Sanitary Association, Berners Street, is just now selling off a quantity of printed cards and leaflets, amongst which you might find what you require.

